# Dynamics of automated body condition scores in grazing Holstein cows in a year-round calving system

**DOI:** 10.3168/jdsc.2024-0619

**Published:** 2024-09-02

**Authors:** C. Hernández-Gotelli, R. Pommiez, F. Aceituno, P. Pinedo

**Affiliations:** 1 Department of Animal Sciences, Colorado State University, Fort Collins, CO 80523; 2 DeLaval S.A., Osorno, Chile 5290000

## Abstract

This observational study aimed to characterize the seasonal dynamics of automated BCS throughout the lactation of Holstein cows in a pasture-based system with year-round calvings. Examining the association between nadir BCS (nBCS; defined as the lowest daily BCS after calving) and peak milk yield within each calving period (calendar seasons equally divided in early and late) was a secondary objective of this research. Retrospective data included 2,164 lactations in 539 primiparous (PRI) and 1,625 multiparous (MLT) Holstein cows that calved from July 2021 to June 2023 in a commercial dairy farm located in Southern Chile. Individual BCS were generated in every milking by an automated BCS camera system. Body condition score at calving (BCSc), nBCS, and the change in BCS from calving to nadir (ΔBCS) were considered for the analysis. Peak milk yield (greatest daily milk yield) values were retrieved from on-farm software and lactations were categorized considering quartiles (Q1 = 25% lowest peak milk yield; Q4 = 25% greatest peak milk yield). Data were examined in PRI and MLT cows separately using multivariable linear models that considered calving period and peak milk yield as the main explanatory variables of interest, whereas BCSc and health status were included as covariables when appropriate. The differences in BCSc among calving periods were more evident in primiparous cows that had the lowest BCSc in early spring (3.13 ± 0.03; ± SE) and the greatest BCSc in late fall (3.51 ± 0.03). In MLT cows, the lowest and greatest BCSc were recognized in late fall (3.07 ± 0.04) and early summer (3.23 ± 0.03), respectively. Only MLT cows had differences in nBCS and ΔBCS across calving periods: The lowest nBCS were identified in late spring (2.71 ± 0.02), early summer (2.71 ± 0.02), and late summer (2.71 ± 0.02). The greatest losses in BCS also occurred in early summer (−0.41 ± 0.02) and in late spring (−0.41 ± 0.02). Time from calving to nBCS varied among calving periods and depended on parity category. Primiparous cows reached nBCS earlier in late summer (65 ± 8 DIM) and late spring (68 ± 7 DIM), whereas MLT had the shortest times to nBCS in late and early fall (67 ± 5 DIM; 68 ± 5 DIM). Greater values at milk peak were associated with lower nBCS only in multiparous cows during the late spring period. We concluded that the dynamics of BCS were associated with calving period in both parity groups. These data could help guide decisions on nutrition and breeding strategies in farms facing comparable conditions.

## Highlights

• As grass-based systems are dependent on the availability of pasture resources year-round, continuous monitoring of the energy balance of cows is crucial.
• Studies exploring the BCS dynamics of grazing dairy cows with year-round calvings are scarce.
• Changes in BCS throughout lactation were associated with calving season in primiparous and multiparous cows.

## Graphical Abstract

**Figure:**
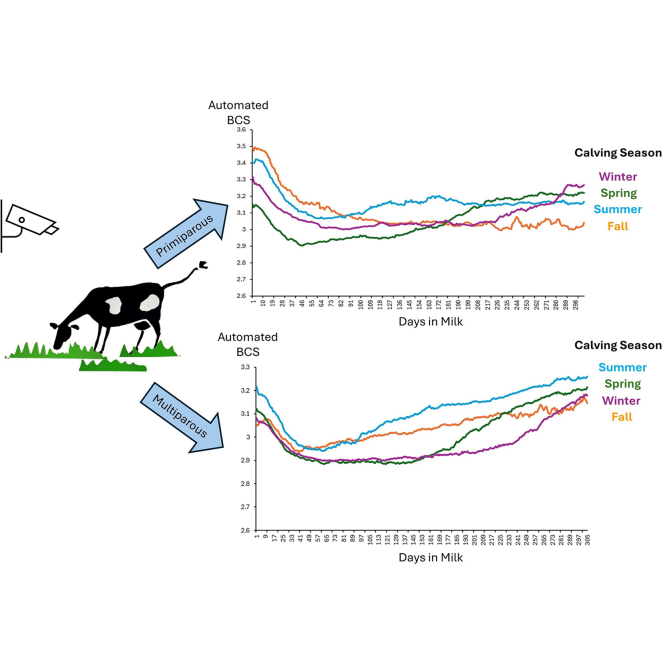
**Summary:** Transversal to most management systems, postpartum dairy cows are challenged by metabolic imbalances resulting from the increased demand for nutrients to initiate lactation, accompanied by slower increments in dry matter intake. This study aimed to characterize the seasonal dynamics of automated body condition score (BCS) throughout the lactation of Holstein cows in a pasture-based system with year-round calvings. The dynamics of BCS throughout the lactation in this Holstein population were associated with calving season in primiparous and multiparous cows. Further research is needed to explore and better understand the effect of seasonality on the BCS of dairy cows maintained on pasture-based systems.

The use of pasture as the main source of nutrients is a common practice in many regions of the world and consumers express a positive perception toward grazing systems associated with sustainable farming practices (USDA, 2022). Universal to most management systems, postpartum dairy cows are challenged by metabolic imbalances resulting from an increased demand for nutrients to initiate lactation, accompanied by slower increments in DMI. The resulting energetic deficit triggers metabolic adjustments favoring the mobilization of fat and labile protein from body energy reserves (Sordillo and Raphael, 2013). As pronounced and extended periods of negative energy balance have multiple detrimental effects, including reduced likelihood of resumption of postpartum ovarian cyclicity (Roche et al., 2009), lowered pregnancy rate (Carvalho et al., 2019), and increased pregnancy loss (Pinedo et al., 2022a), the evaluation of the energy reserves by body condition scoring is a common monitoring practice implemented in dairy farms (Roche et al., 2009).

As pasture-based systems are very dependent on the availability of pasture resources throughout the year, continuous monitoring of the energy balance of cows is crucial. Nonetheless, the ability to precisely investigate the dynamics of BCS is limited when body condition scoring is performed at predetermined time points in the lactation. Recently developed automatic BCS systems provide consistent and more detailed information on the variations of BCS across the entire lactation (Mullins et al., 2019; Pinedo et al., 2022). Moreover, daily body condition assessment allows for the identification of specific landmarks, such as the magnitude of and the time to nadir BCS (**nBCS**; defined as the lowest daily BCS after calving), that previous research has highlighted as associated with cow performance and health (Hernandez-Gotelli et al., 2023; Pinedo et al., 2023). In specific, the aforementioned studies reported that greater milk yield was associated with lower nBCS, whereas cows affected by disease had greater DIM at nBCS in addition to lower nBCS.

System-level and nutrition-related features make the energetic dynamics of grazing transition cows different from those of cows maintained in confinement systems (Roche, 2023). Research suggests that postpartum cows in pasture-based systems lose BCS at a faster rate than cows receiving TMR (Roche et al., 2007). Previous studies have described that, unlike cows in confined systems, pasture cows that calved in spring had a second decline in BCS in mid lactation due to low pasture availability during late summer and fall (Pryce and Harris, 2006; Roche et al., 2007).

As reviewed by Roche (2023), due to the variability of grass growth rate during the year, the season of calving is relevant in the BCS changes occurring throughout the lactation. Notably, because most pasture-based farms group their calvings to take advantage of forage growth curves, the dynamics in BCS for calving periods other than spring have not been widely investigated.

In Chile, 86% of the dairy farms are located in the Southern region, with 75% of the Chilean dairies maintaining cows grazing throughout the year (ODEPA, 2019; Chilean Dairy Consortium, 2024). Remarkably, due to milk price incentives during the winter, a significant proportion of the operations located in Southern Chile have calvings during the whole year. In consequence, the availability of automated BCS systems in commercial farms in this area provides opportunity for exploring the BCS dynamics of grazing dairy cows with year-round calvings.

We hypothesized that calving periods would affect the dynamics of BCS in both primiparous (**PRI**) and multiparous (**MLT**) cows maintained in a pasture-based system with year-round calvings. Therefore, the objective of this study was to characterize the seasonal dynamics of automated BCS throughout the lactation of Holstein cows in a pasture-based system with year-round calvings. Examining the association between nBCS and peak milk yield within each calving period was a secondary objective of this research.

This study used retrospective data from a commercial dairy and the research procedures were approved by the Colorado State University Institutional Animal Care and Use Committee (IACUC) Waiver Subcommittee (protocol #5933). This observational study analyzed data collected from 2,164 lactations (539 PRI and 1,625 MLT) of Holstein cows calving from July 2021 to June 2023 in a grazing dairy located in Puerto Octay, Region de Los Lagos, Chile (latitude: −41.0; longitude: −72.6; altitude: 160 m above sea level). Average minimum and maximum temperatures by season were 5.6°C to 15.9°C (fall), 2.9°C to 10.8°C (winter), 5.3°C to 17. 4°C (spring), and 8.7°C to 23.9°C (summer). Monthly rainfalls were 91.7, 152.4, 53.8, and 43.1 mm for fall, winter, spring, and summer, respectively (Chilean Weather Center, 2024).

The size of the herd varied from 1,000 to 1,150 milking cows during the study period. Lactating cows grazed perennial ryegrass (*Lolium perenne*) and were managed with a daily rotational grazing method using pasture paddocks of varying sizes. Due to the fluctuation in grass growth rates throughout the year, the percentage of grass consumed varied by season with 8 to 10 kg DM/cow in summer, 8 kg DM/cow in winter, 2 to 3 kg DM/cow in fall, and 10 to 12 km DM/cow in spring. The diet was supplemented with corn (30%) and grass (70%) silage offered in feeding barns after milking and concentrate given in the milking parlor, depending on the level of milk production of individual cows. These supplements were offered throughout the whole year, but in variable amounts, depending on grass availability and quality. Cows remained all day at the pasture, except in winter when they were maintained in barns during the nighttime (12 h) to receive supplementary feed. The average milk production per lactation was 7,300 kg/cow and the cows were milked in a 50-unit rotary milking parlor twice per day at approximately 0600 and 1600 h.

Data were collected from calving until dry-off or culling, considering cow as the experimental unit. Cow demographics and health events were extracted from on-farm software (CliWin Software; COOPRINSEM, Osorno, Chile). Milk production and BCS were obtained from DelPro Farm Manager software (DeLaval International AB, Tumba, Sweden). The dataset for the analyses included cow identification number, calving date, lactation number, calving-related and disease events, culling date, and peak milk yield. Information from lactations that started outside the study period was not included. Health events were diagnosed by farm personnel and recorded in the management software when a treatment requiring a milk withdrawal period was administered. Main disorders included retained placenta, metritis, mastitis, pneumonia, lameness, diarrhea, colic, and acidosis. Lactations were categorized by health status as healthy (no-events), with one event, or with multiple events, considering only health disorders occurring before nBCS. For the analysis, cows were categorized into PRI and MLT. Due to the variability of the grass growth rate and management within seasons (Demanet et al., 2015), each calendar season was divided into early (first 45 d) and late (last 45–46 d), resulting in a total of 8 calving periods.

Body condition scores were generated by an automated BCS camera system (DeLaval International AB) validated previously by Mullins et al. (2019) located at the exit of the milking parlor. The BCS calculated by the algorithm was based on the 1 to 5 scale proposed by Ferguson et al. (1994) but was reported in 0.1-point increments. Body condition scores from July 2021 to September 2023 were downloaded from DelPro Farm Manager software. Body condition score at calving (**BCSc**) and nBCS were selected for the analyses, and changes in BCS (**ΔBCS**) were calculated as nBCS − BCSc. Time to nBCS was defined as the DIM when the cow reached nBCS. Peak milk was categorized into quartiles as Q1 (<22.7 kg/d), Q2 (22.7–25.2 kg/d), Q3 (25.3–27.9 kg/d), and Q4 (>27.9 kg/d) in PRI and as Q1 (<28.6 kg/d), Q2 (28.6–31.9 kg/d), Q3 (32.0–35.4 kg/d), and Q4 (>35.4 kg/d) in MLT.

Statistical analyses were performed separately for PRI and MLT cows in R, version 4.2.2 (R Core Team, 2022), using the lmer4, lmerTest, and emmeans packages. Descriptive statistics for BCSc nBCS, ΔBCS, and DIM at nBCS were calculated using ANOVA. Multivariable linear models were fit using BCSc, nBCS, ΔBCS, and DIM at nBCS as the response variables, with calving period as the main predictor. Other fixed effects included in the models were BCSc, peak milk yield, and health status. Potential interactions were tested and removed from the models when *P* > 0.10. Days in milk at the milk yield peak was included as covariable when the association between milk yield peak category and nBCS by calving period was tested. Least squares means were calculated for daily BCS by category of calving period, using repeated measures analysis for linear mixed models, considering cow's ID as a random effect and a compound symmetric/exchangeable correlation with random intercept. The resulting LSM values were presented as BCS curves from calving to 305 DIM (Figure 1). Assumptions of constant variance and normal residuals were assessed using residual diagnostic plots. Results for multivariable linear models are reported in LSM and SE. Statistical significance was established at *P* < 0.05 level using a likelihood ratio test.

**Figure 1:**
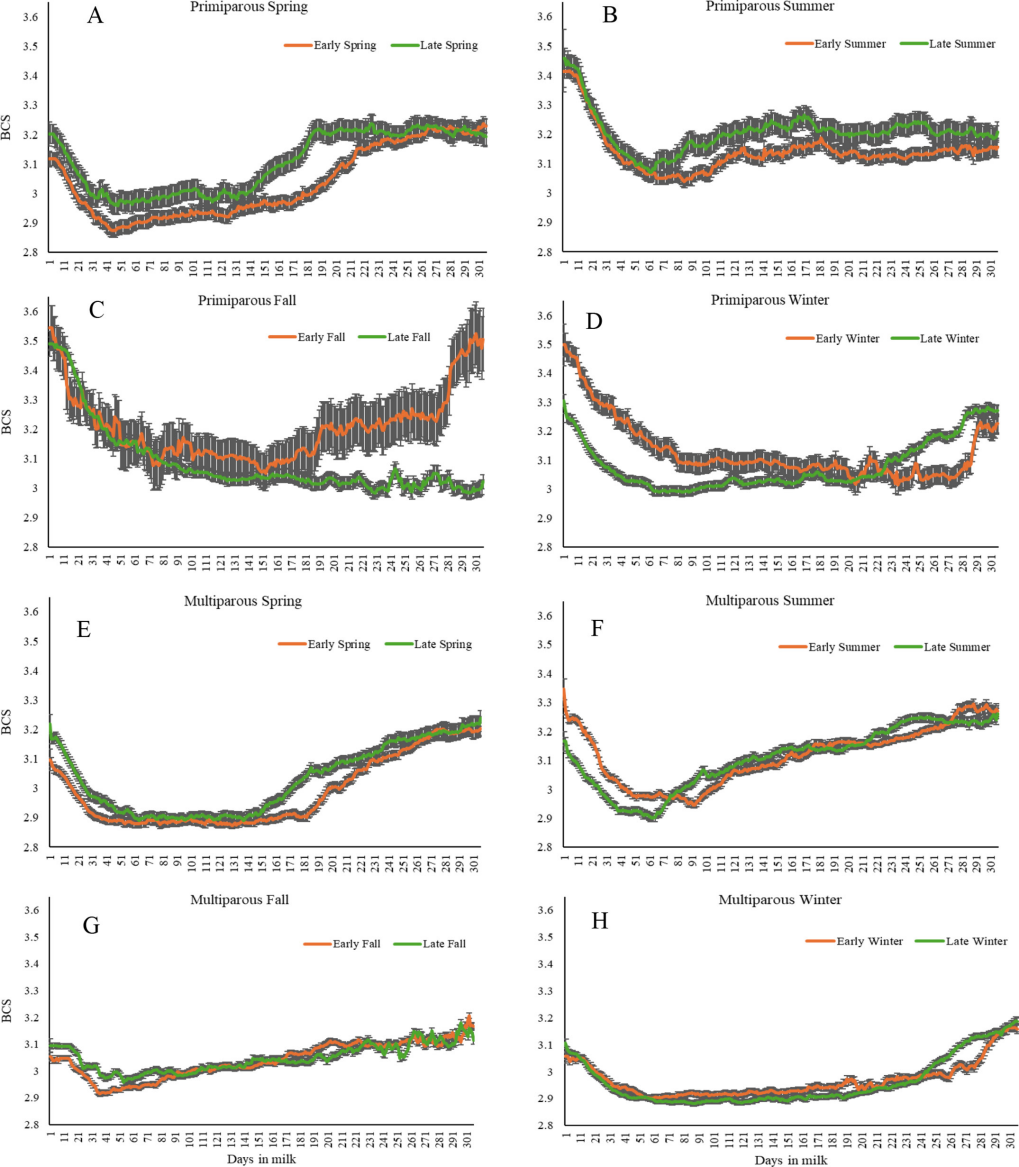
Dynamics of average (±SE) daily automated BCS from calving to 305 DIM by calving period (calendar seasons equally divided in early and late) for primiparous (A: spring, B: summer, C: fall, and D: winter) and multiparous (E: spring, F: summer, G: fall, and H: winter) cows. The linear mixed models for repeated measures considered cow's ID as a random effect for both parity categories.

A total of 2,164 lactations (PRI = 539; MLT = 1,625) and 461,779 daily BCS were included in the analysis. The distribution of calvings by period (early and late) was 7.76% and 11.2% (fall), 9.75% and 21.7% (winter), 16.7% and 11.2% (spring), and 13.1% and 8.64% (summer).

Daily BCS curves by calving period are shown in Figure 1. Among PRI, cows that calved in early spring had the lowest BCS during the first 150 DIM. In MLT, the lowest BCS during the first 150 DIM was identified in cows that calved during early and late spring and early and late winter.

Overall, mean ± SE BCSc was greater in PRI than MLT (3.34 ± 0.01 vs. 3.12 ± 0.01; *P* < 0.0001). Similarly, nBCS was greater in PRI (2.91 ± 0.01 vs. 2.77 ± 0.01; *P* < 0.0001). However, the reduction in BCS until nadir was greater in PRI than in MLT (−0.43 ± 0.011 vs. −0.36 ± 0.006; *P* < 0.0001). In contrast, MLT cows reached nBCS earlier than PRI cows (76 ± 1 DIM vs. 81 ± 2 DIM; *P* = 0.006).

Least squares means for BCSc, nBCS, ΔBCS, and time to nBCS by calving period are reported in Table 1. In PRI cows, the lowest BCSc was in early spring, whereas MLT cows had the lowest BCSc in late fall. For MLT cows, both the lowest nBCS and the greatest ΔBCS between calving and nadir occurred during the summer and during late spring. Primiparous cows reached nBCS earlier in late summer and late spring, whereas MLT reached nBCS in late and early fall.

**Table 1:** Least squares means (SE) for BCS at calving, BCS at nadir, change in BCS (ΔBCS), and time to BCS nadir (nadir DIM) by calving period (dividing calendar seasons in early and late periods) and health status in primiparous and multiparous cows^1^

Calving period	BCS at calving	SE	BCS nadir	SE	ΔBCS	SE	Nadir DIM	SE
Primiparous								
Early fall	3.42abc	0.13	2.94	0.10	−0.34	0.10	91^ab^	21
Late fall	3.51^c^	0.03	2.90	0.03	−0.44	0.03	96^b^	6
Early spring	3.13^a^	0.03	2.86	0.03	−0.48	0.03	74^a^	6
Late spring	3.18^ab^	0.04	2.91	0.04	−0.43	0.04	68^a^	7
Early summer	3.43^c^	0.03	2.89	0.03	−0.45	0.03	81^a^	6
Late summer	3.45^c^	0.05	2.93	0.04	−0.42	0.04	65^ab^	8
Early winter	3.48^c^	0.05	2.97	0.04	−0.37	0.04	87^ab^	8
Late winter	3.30^b^	0.03	2.88	0.02	−0.46	0.02	84^ab^	5
Health status								
Healthy	3.36	0.02	2.93	0.02	−0.42	0.02	76	3
1 event	3.37	0.06	2.92	0.02	−0.42	0.02	81	5
≥2 events	3.36	0.03	2.89	0.05	−0.45	0.05	84	11
Multiparous								
Early fall	3.09^a^	0.03	2.82^bc^	0.02	−0.29^bc^	0.02	68^a^	5
Late fall	3.07^a^	0.04	2.85^c^	0.03	−0.27^c^	0.03	67^a^	5
Early spring	3.10^ab^	0.02	2.72^a^	0.02	−0.39^a^	0.02	90^c^	4
Late spring	3.15^ab^	0.03	2.71^a^	0.02	−0.41^a^	0.02	92^c^	4
Early summer	3.23^b^	0.02	2.71^a^	0.02	−0.41^a^	0.02	87^bc^	4
Late summer	3.10^a^	0.03	2.71^a^	0.02	−0.40^a^	0.02	74^ab^	5
Early winter	3.11^a^	0.03	2.82^c^	0.02	−0.30^c^	0.02	78^ab^	4
Late winter	3.09^a^	0.02	2.76^ab^	0.02	−0.36^ab^	0.02	93^c^	3
Health status								
Healthy	3.12	0.01	2.77	0.01	−0.35	0.01	71^a^	1
1 event	3.14	0.03	2.76	0.02	−0.35	0.02	76^a^	4
≥2 events	3.19	0.05	2.76	0.04	−0.35	0.04	97^b^	8

a–c Different superscripts within columns indicate significant differences (*P* < 0.05).

1 The multivariable linear regression models, for both parity categories (primiparous and multiparous), included calving period, health status, BCS at calving, and peak milk yield as covariables. Interactions tested were not significant and removed from the models.

No differences were identified in BCSc, nBCS, and ΔBCS by health status in PRI or MLT cows. Time to nBCS varied by health status only in MLT, with healthy cows evidencing the earliest nBCS (Table 1).

Milk yield peak means (SE) in PRI were 22.8 (1.2) kg for early fall, 26.4 (0.4) kg for late fall, 28.2 (0.8) kg for early winter, 25.6 (0.3) kg for late winter, 25.7 (0.5) kg for early spring, 25.0 (0.7) kg for late spring, 23.5 (0.4) kg for early summer, and 22.3 (0.7) kg for late summer (*P* < 0.0001). In MLT cows, milk yield peak means were 30.7 (0.4) kg for early fall, 32.8 (0.5) kg for late fall, 32.7 (0.4) kg for early winter, 34.0 (0.3) kg for late winter, 32.3 (0.3) kg for early spring, 32.1 (0.4) kg for late spring, 30.2 (0.4) kg for early summer, and 28.2 (0.5) kg for late summer (*P* < 0.0001). As presented in Figure 2, greater values at milk peak were associated with lower nBCS only in MLT cows during the late spring period.

**Figure 2:**
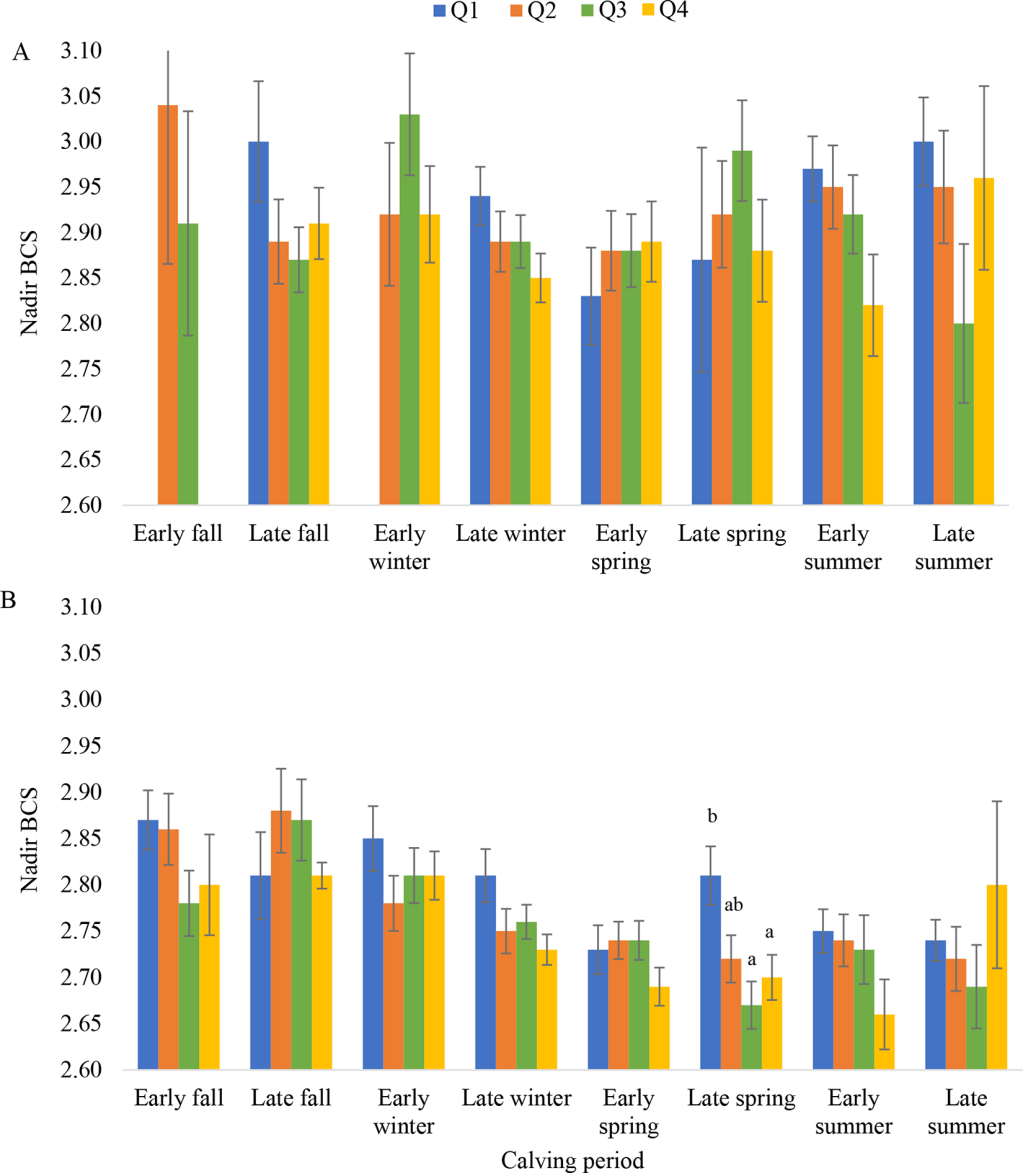
Least squares means (±SE) for BCS at nadir by peak milk yield category (Q1 = lowest milk yield quartile) and calving period in primiparous (A) and multiparous (B) cows. The full model included BCSc and DIM at milk yield peak as covariables. Peak milk yield values were categorized using the quartile distribution (Q1 = lowest milk yield [blue], Q2 = orange; Q3 = green; Q4 = greatest milk yield [yellow]). Note that some calving periods do not have all of the peak milk yield quartiles represented. Different letters within periods indicate *P* < 0.05.

Studies exploring the BCS dynamics of grazing dairy cows with year-round calvings are scarce. The dairy analyzed in the current study represents a considerable proportion of dairies in Southern Chile and allows for the analysis of BCS dynamics of grazing dairy cows with year-round calvings (Chilean Dairy Consortium, 2024).

Due to the effect of precipitation and temperature in the growing cycle of the pasture throughout the year, the amounts of DM available for consumption vary considerably by season. Hence, dairy farms in Southern Chile strategically concentrate the calvings during late winter to early spring and late summer to early fall to meet the highest grass growth rate and the cows' energy demands. Nonetheless, unlike grazing-based dairy systems in New Zealand and Ireland, which concentrate the calving season only in spring (Berry et al., 2006; Roche et al., 2007), due to milk price incentives during the winter in Southern Chile, calvings occur throughout the year.

In agreement with previous research on pasture systems (Berry et al., 2006; Roche et al., 2007), in the current study, PRI cows calved at a greater BCS and had a more moderated nBCS compared with MLT cows. However, PRI cows lost more BCS during their early lactation. Roche et al. (2009) suggested that during the first-lactation cows add to their BCS losses the energy required to grow, causing them to have extended BCS losses throughout their early lactation. Proudfoot and Huzzey (2022) speculated that PRI cows have their feed consumption reduced in early lactation because they experienced more displacements from the feeder by the MLT due to their lower social status, which would reduce their intake of supplements in the feeding barn. The consequences are appreciated in second-parity animals, which evidence the lowest precalving BCS (Roche, 2023). In PRI cows in this specific farm, competition could be a key factor in their postpartum loss of BCS, as these cows were supplemented together with older animals.

Primiparous cows also reached nBCS later in the lactation compared with MLT cows in this and other grazing studies (Berry et al., 2006; Roche et al., 2007). Notably, in confined systems, the opposite has been reported (Truman et al., 2022; Hernandez-Gotelli et al., 2023). In the current study, cows reached nBCS later in their lactation. Due to the differences in feeding and diet management between confined and grazing systems, cows receiving TMR lose BCS at a slower rate than cows on pasture (Roche et al., 2007) and grazing cows require more feed and time to revert the downward trend on their postpartum BCS (Roche, 2023).

In PRI cows, calving period was associated with BCSc but not with nBCS or ΔBCS. However, when BCSc was removed from the nBCS and ΔBCS models the effect of calving period was significant (not shown here), indicating that the effect of BCSc on the BCS losses after calving would be relevant. Time to nBCS was associated with calving period, with later nBCS in PRI cows calved in late (96 DIM) and early (91 DIM) fall. In contrast, in MLT cows, BCSc, nBCS, ΔBCS, and time to nBCS were associated with calving period (Table 1). Multiparous cows that calved during late spring and summer had the lowest nBCS and higher losses in BCS between calving to nadir. This could be explained by the availability of grass and cows' feeding behavior changes during summer. Cows in this geographic area decrease their grazing activity by 17% in summer because they are exposed to heat stress (Arias et al., 2021), and this low DMI during early lactation negatively affects the BCS, increasing BCS losses (Roche et al., 2009). In addition, during summer the grass growth rate is reduced and the vegetative state of the plant changes, decreasing its nutritional value by increasing NDF and reducing ME (Anrique et al., 2008). Thus, early summer seems to be the most challenging period for MLT cows, with the highest BCSc because they ended the previous lactation during spring when the grass had the greatest growth rate and better nutritional value, which decreased significantly during summer.

Although this study did not seek to statistically compare the dynamics of daily BCS by calving period, Figure 1 shows different curve patterns by calving period for PRI and MLT cows. Weather and season effects on the nutrient composition of the grass, environmental conditions, and cows' grazing/feeding behavior affecting DMI (Arias et al., 2021; Roche, 2023) are factors to consider (Berry et al., 2006).

Specific to grazing systems, Roche et al. (2007) described a W-shaped BCS profile on cows that calved during spring, with a second BCS reduction in mid lactation around 122 and 171 DIM. In an earlier study, Berry et al. (2006) reported a plateau profile for BCS until 200 DIM for cows calving during spring. Although these patterns were not evident in our study, PRI cows calving in winter and late fall had extended periods (even reaching 280 DIM) of low BCS after their nBCS. This was most evident in PRI cows calving in late fall that were unable to return to greater BCS. Interestingly, extended periods of low BCS were evident in MLT cows calving in spring, fall, and winter. Notably, BCS curves for PRI calving in spring and MLT calving in summer are similar to those reported in confined systems, with quicker returns to greater BCS after nBCS (Pinedo et al., 2022).

Although the study cows were maintained in a grazing regimen during the whole year, as highlighted by Roche et al. (2007), the BCS patterns can be also affected by the different diet supplementation levels. In our study, MLT cows calving in spring and summer did not recover BCS until they entered a half-day confinement to receive supplementation during fall and winter. Similarly, this half-day confinement could explain the moderated losses in BCS after calving in MLT cows that calved during fall and winter, who had a slower and less pronounced BCS recovery.

In the current study, PRI and MLT cows that calved in early winter and late winter had the greatest milk peak yield. Notably, contrary to previous reports analyzing cows in confinement (Pinedo et al., 2023), only in MLT cows that calved during late spring greater nBCS was associated with lower milk yield (Figure 2).

A limitation of this study is that only health events treated with a product that required a milk withdrawal period were recorded, which likely resulted in an underestimation of the impact of disease on the variables in analysis.

In conclusion, the dynamics of BCS were associated with calving period in both parity groups. Nonetheless, the role of supplementary feeding in the study farm is a factor that should be considered when analyzing the current findings. The information originated from this study could help guide decisions on nutrition and breeding strategies in farms facing comparable conditions.
